# Cue Combination of Conflicting Color and Luminance Edges

**DOI:** 10.1177/2041669515621215

**Published:** 2015-12-28

**Authors:** Rebecca J Sharman, Paul V McGraw, Jonathan W Peirce

**Affiliations:** School of Psychology, University of Nottingham, University Park, UK

**Keywords:** Color, luminance, conflicting, edges, cue combination

## Abstract

Abrupt changes in the color or luminance of a visual image potentially indicate object boundaries. Here, we consider how these cues to the visual “edge” location are combined when they conflict. We measured the extent to which localization of a compound edge can be predicted from a simple maximum likelihood estimation model using the reliability of chromatic (L−M) and luminance signals alone. Maximum likelihood estimation accurately predicted the *pattern* of results across a range of contrasts. Predictions consistently overestimated the relative influence of the luminance cue; although L−M is often considered a poor cue for localization, it was used more than expected. This need not indicate that the visual system is suboptimal but that its priors about which cue is more *useful* are not flat. This may be because, although strong changes in chromaticity typically represent object boundaries, changes in luminance can be caused by either a boundary or a shadow.

Edge perception is fundamental to visual processing; without knowing the locations of visual edges, we could not navigate and interact with the environment. We can easily and accurately localize edges under a variety of conditions, including when vision is challenged, for example, in low light conditions, in a crowded scene, or through a rain-soaked window. The surfaces in natural scenes can be segmented on the basis of both color and luminance information (Fine, MacLeod, & Boynton, 2003; Hansen & Gegenfurtner, 2009; Johnson, Kingdom, & Baker, 2005) such that either cue might be used to detect an object boundary.

If changes in chromaticity and luminance occur and are sufficiently displaced then two edges are perceived, but if they are spatially proximal and cooriented then a single edge is perceived; the two signals are somehow combined to form a single estimate of edge location. The rule that is used for this combination, however, is unknown. It is unclear, for instance, whether the two cues contribute equally in edge localization tasks and what happens when they conflict.

When color and luminance cues conflict, there are several possible ways that edge location could be determined as both chromatic and luminance information can be used to make edge localization judgments. When contrast is equated in multiples of detection threshold, Vernier thresholds are not significantly different for isoluminant and achromatic stimuli (Krauskopf & Forte, 2002). Similarly, the presence of a luminance flanker does not have a greater effect on edge localization than a chromatic flanker, again, if performance has been equated for the cues alone (Rivest & Cavanagh, 1996). These studies suggest that, if the cues are equated in perceptual intensity, they should have equal influence on edge perception.

On the other hand, there are many visual illusions that offer examples of luminance information dominating chromatic information. In the Boynton illusion, straight chromatic edges appear to follow the contours of curved luminance lines (Kaiser, 1996). Chromatic after-images also appear to follow achromatic information, as demonstrated by the Big Spanish Castle illusion (Sadowski, 2006). In this illusion, adaptation to a chromatic negative image followed by presentation of an achromatic version of the original image leads to perception of a sharp, normally colored version of the image. Note that, if a blank field is presented instead of the achromatic image, the chromatic after-image on its own is blurry and indistinct. Similarly, in the Watercolor Illusion, chromatic filling-in appears to be constrained by luminance boundaries (Pinna, Brelstaff, & Spillmann, 2001). Lastly, luminance information has also been shown to constrain chromatic blur in natural scenes (Sharman, McGraw, & Peirce, 2013). In all of these examples, luminance information appears to constrain the percept of chromatic edges.

In other circumstances, however, chromatic signals appear to dominate, masking luminance signals. Kingdom, Bell, Gheorghiu, and Malkoc (2010) measured the relative salience of chromatic versus luminance suprathreshold modulations when these were either separated in time or presented simultaneously at orthogonal orientations to create a chromatic-luminance checkerboard. They found that the salience of the luminance signal decreased relative to the chromatic signal when the two stimuli were presented simultaneously, which they attribute to masking. This study differed from those above in a number of ways, however; the modulations in luminance and color were always orthogonal in orientation, the contrasts used were comfortably suprathreshold, and the task required the participant to report which component was the higher contrast. It is unclear which of these characteristics caused color to mask luminance in the compound condition rather than luminance masking chromatic signals, as might have been expected.

These examples demonstrate that both luminance and L−M information can dominate under different circumstances. In the case of localization of conflicting edges, it is unclear whether the cues will exert equal influence or whether one will dominate the other. Here, we have addressed the question in a Bayesian framework in which we have measured the reliability of each cue alone (the variability of participants’ judgments of edge location) and then measured the perceived position of a compound edge for which the two components have slightly different locations. Critically, the offset between the L−M and luminance component is small enough that they are fused perceptually and appear as a single edge. From a maximum likelihood estimation (MLE) model, we predicted where the compound edge might be perceived. The MLE takes into account the reliability of the two cues; the more reliable the cue, as measured in our case by the variance in localization judgments, the greater weight it should be given in the judgment. It should be noted that, as the name suggests, the MLE is concerned only with the *likelihood* function of the Bayesian estimation; it has a flat *prior* about their expected utility. MLE has successfully predicted cue combination in several visual areas including combination of texture and disparity in judgments of slant (Hillis, Watt, Landy, & Banks, 2004), auditory and visual cues in the ventriloquist effect (Alais & Burr, 2004), and stereo and texture information in judgments of slant (Knill & Saunders, 2003).

In piloting, we equated, approximately, the reliability of the two cues by manipulating their contrast. As a result, the predicted percept of the compound edge is centered roughly between the physical locations of our L−M edge and our achromatic edge (Figure 1). From this point, manipulating the contrast of the L−M component alters the reliability of that cue, which shifts the predicted location of the compound edge (the predicted location has a positive slope in Figure 1). The model accurately predicted the *pattern* of results across contrasts (data shown in the solid line). However, it consistently overestimated the relative importance of the luminance cue. Participants’ judgments were consistently closer to the L−M edge than MLE predicted.

**Figure 1.**
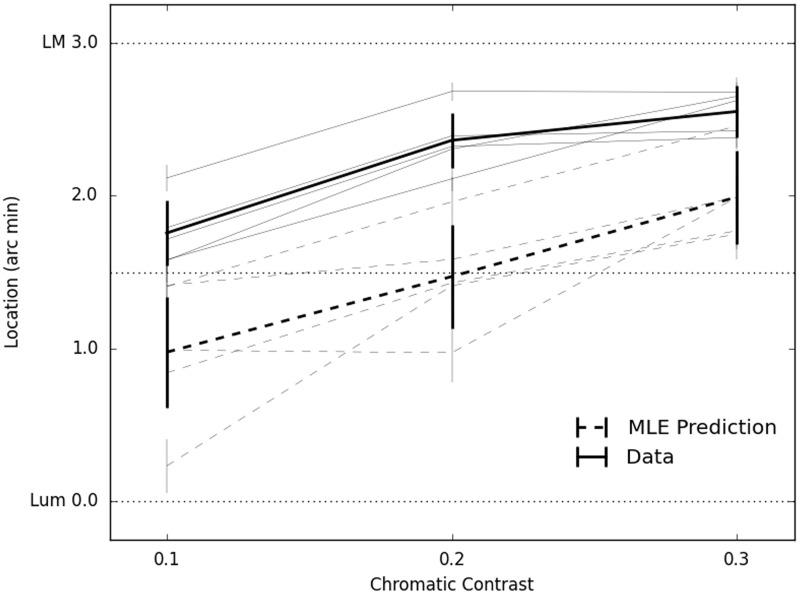
Location of the combined edge according to MLE predictions based on individual cues (broken lines) or based on percept of the compound edge (solid lines). Individual observers’ data were shown in gray and group means are shown in bold black. The veridical position of the luminance edge is at 0.0 arc min on the abscissa and the chromatic edge is depicted at 3.0 arc min. The fact that the MLE prediction crosses the veridical center at a chromatic contrast of 0.2 indicates that the two cues were successfully equated in contrast/reliability at this point. However, the data show that participants are judging the edge to be closer to the chromatic edge than predicted. Error bars represent ±1 standard error of the mean (for individuals, this was calculated from bootstrapped analysis and for the group, this is the standard error of the mean between individuals).

It appears that the weights generated from measurements of each component in isolation are not sufficient to predict edge localization in conflicting conditions. The L−M component is given a greater weight than would be predicted by its reliability alone. The fact that the predictions did not match the behavior of participants suggests either that the visual system is failing to combine the signals in an optimal manner, in a Bayesian sense, or that the system is optimal but using additional information. Since MLE is based on optimizing the *likelihood*, but assumes a flat *prior*, we might presume that it is this prior that is favoring the chromatic cue. We wonder, then, from where this prior for using L−M information arises.

Although luminance has higher effective contrast in natural scenes (Rivest & Cavanagh, 1996) and is a more *reliable* cue in most natural viewing conditions, changes in luminance can also represent variations in lighting, such as cast shadows. This could potentially make L−M information a more *useful* cue for detecting object borders (McGraw, Whitaker, Badcock, & Skillen, 2003; Ruderman, Cronin, & Chiao, 1998). If one cue consistently represents object boundaries, whereas the other can represent either object boundaries or changes in lighting, then it might well be that the visual system has developed a Bayesian prior for edge localization that favors the chromatic information. Although we are unaware of data from natural image statistics that have measured the expected priors for chromatic and luminance edge location, this explanation seems best placed currently to explain a set of results that, to us, were somewhat surprising initially.

## Methods

### Participants

Three male and two female volunteers (including the first author), with normal or corrected-to-normal vision, gave their informed consent to participant in the study. All procedures were approved by the School of Psychology Ethics Committee, University of Nottingham, UK and were in accordance with the Helsinki Declaration (2008, Version 6).

### Apparatus

A gamma-corrected computer-controlled cathode-ray-tube monitor (Iiyama, Vision Master Pro 454, resolution: 1024 × 768, refresh rate: 85 Hz) was used to present stimuli. A chin rest was used to give a constant viewing distance of 367 cm. At this distance, each pixel subtended 0.006 degrees of visual angle.

### Stimulus Generation

Two bipartite edges were created in MB-DKL space (Derrington, Krauskopf, & Lennie, 1984; Macleod & Boynton, 1979), one comprising luminance information (L + M) and one comprising chromatic information (L−M). Chromatic contrast values are specified as fractions of the maximal modulation along the L−M cardinal axis in MB-DKL space.

Individual differences in isoluminant plane (resulting in a luminance artifact for the L−M stimulus) might serve to reduce the effects that we were measuring. We, therefore, measured these for each observer and corrected for them in stimulus presentation. Measurements were made by minimizing the artifactual motion signal that arises when the chromatic stimulus is not isoluminant for an individual (Anstis & Cavanagh, 1983). In this method, two chromatic gratings are interleaved with two chromatic low-contrast gratings. All had spatial frequency of 2 cpd and were presented in Gaussian envelopes with 2° diameter (to 3 *SD* of the Gaussian). The achromatic Gabors were presented at 0.1 Michelson contrast and the chromatic Gabors were presented at full contrast. The phase of the four gratings is arranged in quadrature (e.g., chromatic gratings at 0 and π phase, with the achromatic gratings at π/2 and 3π/2). Note that when the channels are independent the stimulus is simply counterphasing, but if the chromatic channel is carrying a luminance artifact then a phase advance (i.e., motion) will be perceived in one direction or the other (depending on the direction of the luminance artifact on the chromatic grating). Participants reported, in a two-alternative forced force, the perceived direction of any motion while the actual phase advance was randomized in its direction. The elevation of the chromatic stimulus was then adjusted using a one-up, one-down staircase procedure until no coherent motion was perceived. The elevations generated using this procedure were then applied as deviations from photometric isoluminance in MB-DKL space. The deviations for the participants were −2.539°, −3.384°, −2.667°, −4.456°, and −3.478°.

The edges were Gaussian blurred (σ = 0.1°) and 4.5° × 1° in size. The compound stimuli were created by summing the relevant component edges together. The achromatic (luminance only) edges were presented at a Michelson contrast of 0.02, and the isoluminant edges (chromatic only) were presented at contrasts of 0.1, 0.2, and 0.3. Note that in this color space, these values are, essentially, arbitrary. They reflect simply fractions of the maximal modulation in each direction. The values were chosen so as to equate roughly the reliability of the cues in isolation; with contrasts of 0.1 versus 0.02, the luminance cue was more reliable, but with contrasts of 0.3 versus 0.02, the chromatic cue was more reliable. By equating the cues in this way, the MLE predicts that the perceived edge should be half-way between the chromatic and luminance boundary (the dashed line prediction falls at a location of 1.5 for this middle contrast). A vertical marker, with a width of one pixel, was presented immediately below the edge, at a random horizontal offset from the initial edge position on each trial.

**Figure 2.**
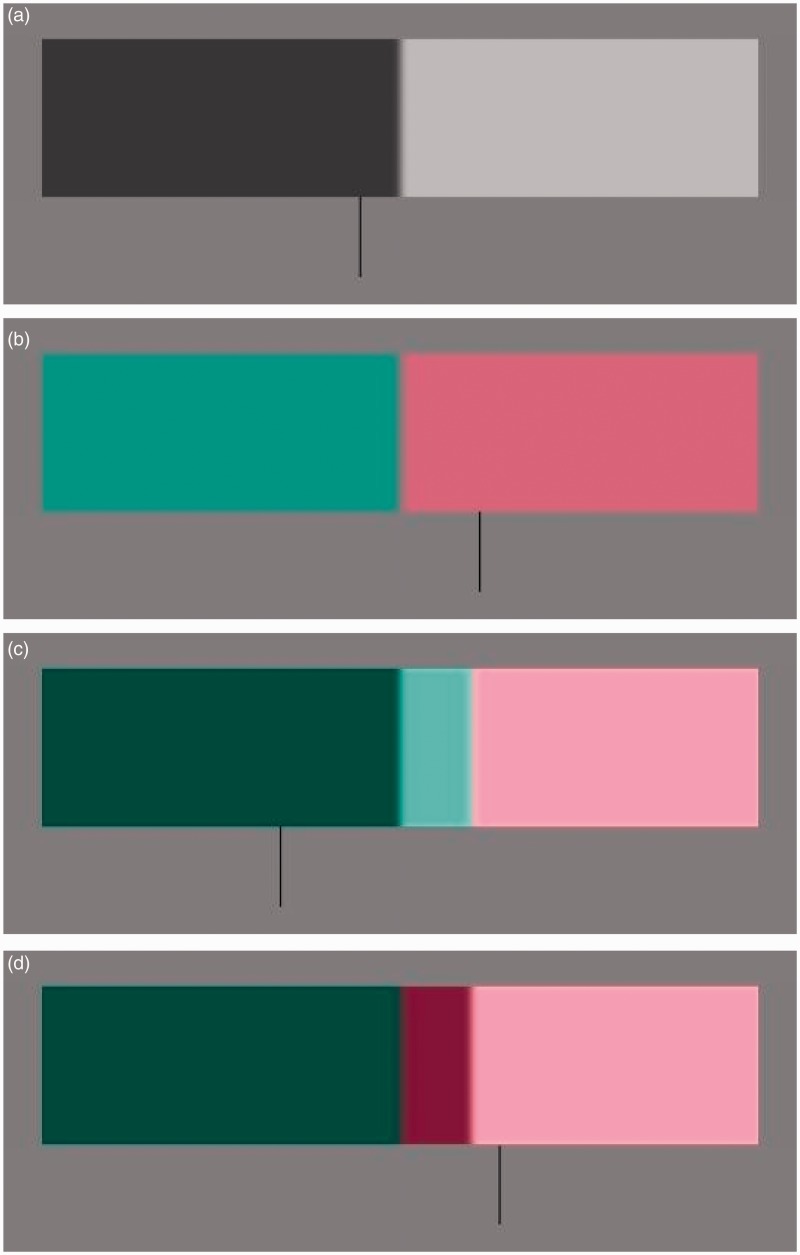
Example stimuli. (a) The luminance information alone condition. (b) The chromatic information alone condition. (c, d) Examples of compound stimuli. The gap between the chromatic and luminance information has been substantially exaggerated for illustration; in the actual stimuli, the edges appeared fused and the offset between components was not perceptible.

Examples of the stimuli used in both the alone and combined conditions are shown in Figure 2. For the compound stimuli, we tested conditions in which the higher luminance was combined with the L (pink) side of the chromatic edge and when it was combined with the M (green) side and reflections of these combinations, although we saw no indication that this affected the data. This counterbalancing also served to account for the potential confound that edge locations appear shifted toward the darker side of a luminance edge (Georgeson & Freeman, 1997). Similarly, a vertical edge presented on a CRT monitor (as was the case here) could appear shifted in its location as a result of uneven slew rate of the monitor (taking different periods for the electron beam to switch from dark to light than from light to dark). In our case, the low contrast and blur of the edges means that the rate of change from one pixel to the next (either increasing or decreasing) was very small, and any potential effect of slew rate would, therefore, be extremely small. Furthermore, the fact that measurements were made with counterbalanced transitions, as described earlier, mean that this type of artifact cannot account for the shifts we have observed. The effect we observed favored the chromatic edge location irrespective of whether that was on the darker or lighter side of a luminance boundary.

### Procedure

Participants were presented with the edge and marker and used the mouse to move the edge until they were satisfied that the two were aligned. By moving the bipartite edge (which had been Gaussian-blurred) rather than the bar, we were able to measure the reliability more precisely than a single pixel’s width. There was no limit to presentation time and the participant’s response began the next trial, following a 300 ms ISI. Presentation order of the conditions was randomized and 40 trials were collected per participant, per condition.

### Maximum Likelihood Estimation

We used the MLE model, as outlined in Hillis et al. (2004), to generate cue combination predictions. The method is described in detail below.

We combined unbiased estimates of edge location based on color (${\overset{\wedge }{S}}_{\mathrm{col}}$) and luminance (${\overset{\wedge }{S}}_{\mathrm{Lum}}$) with variances $\sigma_{\mathrm{col}}^{2}$ and $\sigma_{\mathrm{Lum}}^{2}$, respectively, to produce a prediction that maximizes the Bayesian likelihood function

(1) $$\overset{\wedge }{S}=\omega_{\mathrm{Col}}{\overset{\wedge }{S}}_{\mathrm{Col}}+\omega_{\mathrm{Lum}}{\overset{\wedge }{S}}_{\mathrm{Lum}}$$

where the weights are given by

(2) $$\omega_{\mathrm{Col}}=\frac{r_{\mathrm{Col}}}{r_{\mathrm{Col}}+r_{\mathrm{Lum}}}\;\text{and}\;\omega_{\mathrm{Lum}}=\frac{r_{\mathrm{Lum}}}{r_{\mathrm{Lum}}+r_{\mathrm{Col}}}$$

The reliability measures (*r_Col_* and *r_Lum_*) are simply the inverse of the respective variances. These variances were calculated from the distances between the participant’s judgments and the veridical edge location across the 40 trials for each condition. Occasionally, participants would push the mouse button accidentally before they had finished aligning the edge with the marker. As a result, outliers, defined as having a z-score greater than 3.0 or less than −3.0, were removed prior to the calculation of this variance value.
